# Development and Evaluation of a Visual Remediation Intervention for People with Schizophrenia

**DOI:** 10.20900/jpbs.20200017

**Published:** 2020-07-20

**Authors:** Steven M. Silverstein, Aaron R. Seitz, Anthony O. Ahmed, Judy L. Thompson, Vance Zemon, Michael Gara, Pamela D. Butler

**Affiliations:** 1 Department of Psychiatry, University of Rochester Medical Center, 300 Crittenden Boulevard, Rochester, NY 14642, USA; 2 Departments of Neuroscience and Ophthalmology, University of Rochester Medical Center, 210 Crittenden Boulevard, Rochester, NY 14620, USA; 3 Department of Psychiatry, Robert Wood Johnson Medical School, Rutgers University, 671 Hoes Lane West, Piscataway, NJ 08854, USA; 4 Department of Ophthalmology, Robert Wood Johnson Medical School, Rutgers University, 675 Hoes Lane West, Piscataway, NJ 08854, USA; 5 Department of Psychology, University of California at Riverside, 900 University Avenue, Riverside, CA 92521, USA; 6 Department of Psychiatry, Weill-Medical College of Cornell University, 21 Bloomingdale Road, White Plains, NY 10605, USA; 7 New York Presbyterian Hospital, 21 Bloomingdale Road, White Plains, NY 10605, USA; 8 Department of Psychiatric Rehabilitation and Counseling Professions, Robert Wood Johnson Medical School, Rutgers University, 75 Hoes Lane West, Piscataway, NJ 08854, USA; 9 Clinical Research Department, Nathan Kline Institute for Psychiatric Research, 140 Old Orangeburg Road, Orangeburg, NY 10962, USA; 10 Department of Psychiatry, New York University Langone Medical Center, 550 First Avenue, New York, NY 10016, USA; 11 Ferkauf Graduate School of Psychology, Yeshiva University, 1225 Morris Park Avenue, Bronx, NY 110461, USA

**Keywords:** schizophrenia, perception, perceptual remediation, vision, cognitive remediation, rehabilitation, contrast sensitivity, perceptual organization

## Abstract

It is now well documented that schizophrenia is associated with impairments in visual processing at all levels of vision, and that these disturbances are related to deficits in multiple higher-level cognitive and social cognitive functions. Visual remediation methods have been slow to appear in the literature as a potential treatment strategy to target these impairments, however, in contrast to interventions that aim to improve auditory and higher cognitive functions in schizophrenia. In this report, we describe a National Institute of Mental Health (NIMH)-funded R61/R33 grant that uses a phased approach to optimize and evaluate a novel visual remediation intervention for people with schizophrenia. The goals of this project are: (1) in the R61 phase, to establish the optimal components and dose (number of sessions) of a visual remediation intervention from among two specific visual training strategies (and their combination) for improving low and mid-level visual functions in schizophrenia; and (2) in the R33 phase, to determine the extent to which the optimal intervention improves not only visual processing but also higher-level cognitive and role functions. Here we present the scientific background for and innovation of the study, along with our methods, hypotheses, and preliminary data. The results of this study will help determine the utility of this novel intervention approach for targeting visual perceptual, cognitive, and functional impairments in schizophrenia.

## INTRODUCTION

This paper describes a sequence of two clinical trials utilizing the NIH (NIMH) R61/R33 grant mechanism. With this type of grant, the goals of the initial clinical trial (the R61) are to determine the ability of an intervention to successfully change a specific “target” mechanism, and to optimize the intervention through the evaluation of specific intervention parameters (e.g., dose, intervention components). The aim of the second clinical trial (the R33) is then to determine if the optimal intervention not only affects the “target” but also generalizes to other functional outcomes. The goals of this specific project are: (1) in the R61 phase, to evaluate the effects of two visual remediation strategies (and their combination) on the target of visual perception in schizophrenia in order to determine which approach, and at what dose, most effectively drives gains in the targeted perceptual processes in schizophrenia, and (2) in the R33 phase, to evaluate the effectiveness of the “optimal” intervention from the R61 in a new and larger sample, and to test the link between visual target engagement and treatment-related changes in higher-order cognitive, social cognitive, and functional domains. If successful, this project will identify a novel intervention strategy that fills the unmet need of alleviating core visual perceptual deficits and related higher-order cognitive and functional impairments in schizophrenia.

## SIGNIFICANCE

It has become increasingly clear that schizophrenia is associated not only with cognitive deficits, but also with a wide range of visual perceptual impairments. These include alterations in low-level vision, such as visual acuity and contrast sensitivity [1–5]; mid-level vision, including various aspects of perceptual organization [6–10]; and high-level vision, such as in the effects of prior knowledge on visual perception [11,12]. It has been shown that in many cases, these visual processing impairments are not manifestations of a generalized deficit, and are independent of medication effects [10,13–15]. Furthermore, some of these visual perceptual deficits have been shown to predate psychosis onset [16–18]. Multiple studies have indicated that these visual perceptual deficits are significantly related to impairments in higher-level cognitive and social cognitive functions, such as visual working memory [19–23], object recognition [7], and facial emotion recognition [24], as well as to poorer functional outcomes [25,26]. In addition to laboratory demonstrations of impairment, approximately 2/3rds of individuals with schizophrenia report visual perceptual abnormalities [27–29], and these distortions are among the strongest predictors of conversion to schizophrenia-spectrum disorders among high-risk youth [17]. The rate of visual hallucinations in schizophrenia has been estimated to be approximately 27% [30], with the prevalence among those with chronic schizophrenia closer to 50% [31,32]. It has also been shown that visual hallucinations are often under-reported due to a lack of careful questioning about them during clinical assessments [31]. Consistent with this, an experience-sampling study reported a rate of visual hallucinations of 62.5% in people with schizophrenia [33].

An open question is the extent to which some or all of these anomalies are related. As noted above, low-level visual disturbances are related to higher level visual impairments in schizophrenia, and there is some data suggesting that they are related to subjective experiences such as visual distortions [13,34] and possibly to visual hallucinations [32] as well (e.g., via attempts to compensate for reduced or degraded retinal and visual cortex input, as has been found in other disorders [35–38]). Consistent with the idea that living with abnormal visual representations and their consequences (e.g., slowness in, or interference with activation of appropriate lexical codes [39–41]) is related to the emergence of psychotic symptoms, evidence indicates that abnormal vision in childhood is related to an increased risk for schizophrenia [42–44], and that being born blind (but not acquired blindness) has a protective effect against the development of schizophrenia [43,45–47]. In short, visual disturbances are common in people with schizophrenia, they can have significant downstream effects, and these effects are often not subtle.

Despite this growing literature regarding the variety, prevalence, and significance of visual perceptual impairments in schizophrenia, few studies have evaluated the therapeutic potential of interventions designed to target specific visual processes to improve visual and related functions in this condition. Initial efforts to do so have demonstrated improvements on trained [48–50], as well as untrained [48,50] tasks. Although promising, these studies have generally been small, lacked a control group, and used training paradigms with a limited number of sessions. At the same time, a growing number of perceptual learning studies indicate that in both healthy controls and people with schizophrenia [51–56], performance on visual tasks can be improved with repeated practice over relatively short periods (e.g., 1–5 days). For example, we previously published a review on improvements in visual perceptual organization in schizophrenia after repeated task performance (typically over several consecutive days), based largely on a number of our earlier contour integration studies [56]. Furthermore, studies of visual interventions implemented with non-psychiatric samples, including the contrast sensitivity training program we are using for the current project, have demonstrated treatment-related gains that extended beyond the trained visual function, including improved reading skills in controls [52] and people with dyslexia [57–59], and improved batting averages in college baseball players [60]. These results suggest that improvements in low-level visual processes such as contrast sensitivity can lead to gains in higher-level, real-world functions.

Finally, a body of work evaluating the effects of a cognitive training program that emphasizes auditory sensory processing has demonstrated significant treatment-related improvements in higher-level auditory and verbal functions in schizophrenia, such as verbal working memory and verbal learning [61–63]. Although this program includes training on both low-level auditory targets and higher-level functions such as verbal memory and learning, it was observed that training-related gains in speed of auditory processing, a specific perceptual target of this intervention, were associated with improvements in global cognition, suggesting that *gains in lower-level perceptual processes may contribute to improvements in higher-order cognitive functions in schizophrenia* [64]. In short, multiple lines of evidence converge to suggest that remediation of visual function is possible, and that it could lead to gains in higher cognitive and related functions. However, despite this promise, and despite visual remediation being a well-developed field in its own right, e.g., in the areas of neurology and ophthalmology [65–67], what is not known is whether, in a well-controlled study with a sufficient sample size and duration of treatment: (a) visual processing can be significantly improved in schizophrenia; and (b) improvements in visual processing will lead to improvements in higher-order cognition, social cognition, and functional capacity. Therefore, we sought to address these questions by evaluating a focused and systematic training program to remediate the two core processes identified by the NIMH-funded CNTRICS (Cognitive Neuroscience Treatment Research to Improve Cognition in Schizophrenia) initiative as being involved in visual impairments in schizophrenia: *gain control* and *integration* [68]. Gain control refers to the homeostatic amplification or attenuation of signals to keep neural activity within an adaptively limited signaling range, which serves to prevent under- or overstimulation, respectively. Integration refers to the binding of related stimulus feature representations into higher order representations for later processing (e.g., grouping by collinearity and similarity). For this project, we are focusing on what are arguably the two most frequently demonstrated manifestations of gain control and integration failures in vision in schizophrenia: impairments in *contrast sensitivity* [1,21,24,68–72] and *perceptual organization* [7,8,10,68], respectively. To operationalize our treatment targets we are using established tests of contrast sensitivity (CS) and perceptual organization (PO). To determine the specific effects of training in each of these processes individually and in combination, we set out to evaluate two complementary forms of visual remediation - one designed to target CS, and the other designed to target PO.

CS and PO have been extensively studied in schizophrenia, their neural mechanisms are relatively well-understood (see below), and their impairments predict higher-level dysfunction. For example, impairments in CS have been demonstrated in psychophysical, electrophysiological, and brain imaging studies of schizophrenia [1,21,24,69–71], and these are associated with failures in the later process of PO, as elaborated upon below, as well as with poorer facial emotion recognition [24], reading ability [19,20], cognition [21], and community functioning [1], in addition to perceptual distortions [34] in this condition. Furthermore, our preliminary data (see below) and evidence from the ophthalmology literature [73] indicate an association between impairments in CS and slower speed of processing, suggesting that impaired CS may affect performance on speed of processing tasks that use visual stimuli, and/or that both emerge from a similar neural substrate. It is also possible that reduced speed of processing leads to impaired CS, but we consider this unlikely due to the wealth of evidence on impaired CS in schizophrenia on tasks without a timed component.

PO impairments in schizophrenia have been observed in over 50 laboratory studies (reviewed in [7,8,10]), including in psychophysical [10], ERP [7,74] and fMRI investigations [7,75,76]. Subjective visual integration difficulties have also been reported in the clinical literature, an example of which is: “I have to put things together in my head. If I look at my watch I see the watch, the watchstrap, face, hands, and so on, then I have to put them together to get it into one piece” [77] (p. 229). As with CS, PO deficits are associated with decrements in higher-level functions, including constructing visual representations such as faces from degraded stimuli [4,78], forming visual memory representations [22,23], and recognizing facial emotional expressions [79]. PO impairments are also related to poorer functional outcomes [80,81]. For example, abnormal visual PO negatively predicted discharge to the community from a long-term inpatient psychiatric rehabilitation program more strongly than neuropsychological measures of reaction time, attention, memory, and executive functioning, over a 3-year period [82]. Thus, impairments in both CS and PO have been related to poorer role functioning [25,26]. Consistent with this, structural equation modeling (SEM) studies indicate a single pathway from visual dysfunction to functional outcome, with mediating variables that include social cognition [25,26,83]. Such findings support a cascade model in which degraded visual representations contribute to difficulties in higher-level processing [25,84], and “helps to provide a rationale for early perceptual and cognitive interventions, such as plasticity-based training,” [85] (p. 1223).

The neural mechanisms involved in CS are relatively well understood. As noted above, CS has been viewed as a form of gain control [86,87]. Gain control involves both amplification and suppression of signals to keep neural activity at or near optimal levels; this homeostatic function thereby serves to reduce the risk of sensory deprivation-induced hallucinations that may result from insufficient cortical activation [88], and the risk of stimulus overload related to excess activation [68,87,89]. Weaker signals, such as those involving low contrast and/or low spatial frequency (LSF), are amplified, and stronger signals are attenuated, with the full contrast-response function therefore following a classic sigmoidal curve. At the neural level, both amplification and attenuation are thought to arise from divisive normalization [90–92], in which target signal strength is modulated as a function of total activation in the cortical region [1,24,93–96]. Evidence that observer CS in a psychophysical task corresponds with level of neural activation, and that it is modulated by gain control, comes from several sources. One is that, using single-unit microelectrode recording in cat V1, the inverted U-shaped psychophysical CS function (CSF) was highly correlated with the neuronal CSF [97,98]. Another is that the fMRI BOLD response in V1 in humans covaries with contrast enhancement [98] and spatial frequency (SF) [99], with the relationship being especially tightly coupled for LSF stimuli [100]. In short, gain control keeps responses within an adaptively limited signaling range, and both of the CS assessment formats we propose to use generate data that can be interpreted clearly within cognitive neuroscience models of gain control in schizophrenia and healthy populations.

The neural mechanisms involved in PO are also relatively well-understood. Grouping of visual features that are collinear, or whose orientation changes in a systematic way so as to form a surface that might be found in nature (e.g., a curve) involves short-range lateral excitatory activity between neurons signaling correlated features, and corresponding inhibitory activity between neurons signaling visual features whose orientations are not strongly correlated [14,101–104]. Feedback to primary visual cortex (area V1) from higher visual regions associated with shape perception (and with larger receptive fields), such as V2, V3, V4, and the lateral occipital complex (LOC) strengthens activation related to lines, surfaces, and shapes relative to background features [105,106]. In cases where configurations are more novel, feedback from frontal regions may be needed to reliably perceive perceptual wholes [107].

### Training and Assessing CS

As noted, the first level of remediation that we will evaluate targets gain control, in the form of CS. To remediate CS impairments in schizophrenia, we are using the contrast sensitivity training (CST) program of the Sightseeing App, which targets CS across a wide range of spatial frequencies (i.e., fine to broad lines corresponding to high to low SF, respectively). Sightseeing is ready for early-phase testing in participants with schizophrenia, and has been made available by the University of California, Riverside Brain Game Center for Mental Fitness and Well-being that was established by the second author [52,60,108]. This intervention is described more fully below.

To assess target engagement for the CS intervention (CST), we will use two complementary paradigms, one psychophysical and the other electrophysiological, both of which were recommended by the CNTRICS initiative to assess the construct of gain control in vision [86,109]. *Psychophysical CS* assesses the lowest level of contrast at which stimuli presented at different spatial frequencies (SFs) can be detected. Studies of psychophysical CS show that people with schizophrenia need higher contrast than controls to detect stimuli across the range of SFs (i.e., they have lower CS overall), although deficits in processing *low* SF (LSF) stimuli have been more pronounced in some studies of schizophrenia [1,21,24,110]. Furthermore, deficits in LSF processing are related to impairments in object recognition [110], face processing, and facial emotion recognition in schizophrenia [4,78,111–116]. Therefore, we are focusing primarily on CS for LSF stimuli, although we will also examine effects for high SF (HSF) stimuli, given that some studies indicate a schizophrenia-related impairment in HSF processing as well [117,118]. Because features within LSF stimuli are relatively large, there is less of an effect of visual acuity on their detection, as opposed to fine-grained HSF stimuli, the detection of which relies rely heavily on acuity. Thus, our focus on LSF processing also helps to minimize the potential confound of impaired visual acuity in people with schizophrenia on task performance [5,117,119]. *Electrophysiological CS* involves recording the steady-state visual evoked potential (ssVEP) in response to stimuli varying in contrast [1,86,95,96,109]. The ssVEP measure provides a rapid, objective assessment of visual cortical responses to a range of contrast levels *without requiring behavioral responses from participants*. Studies of ssVEP in schizophrenia consistently show impairments, and these are most pronounced at low contrast levels. These investigations have also indicated that weaker ssVEP responses to low-to moderate contrast stimuli (16–32%) are significantly correlated with poorer behaviorally-assessed CS [1], facial emotion recognition [24], Global Assessment of Functioning (GAF) scores, and Problem Solving Factor scores on the Independent Living Scale [1].

### Training and Assessing PO

The second level of remediation in the R61 targets visual integration, in the form of PO. We are focusing specifically on the visual PO process of contour integration. We have developed a program for contour integration training (CIT), also built into the Sightseeing App, via modification of a contour integration task developed for use in schizophrenia by the first author and colleagues, and used previously in multiple behavioral, ERP, and fMRI studies [8,74–76,117,120]. Our hypothesis regarding the ability of CIT to drive gains in PO is based on prior demonstrations of perceptual learning in controls and participants with schizophrenia (albeit at a slower rate in the latter group) with similar versions of this task [8,56,80]. For example, healthy controls showed improved detection of a collinear path over 12 days of training [121], improved performance on a closed contour integration task across test sessions that spanned 2 consecutive days [122], and gains in identifying interpolated shapes over 4 days [123]. In addition, in monkeys, behavioral performance and V1 activity increased consistently over 10 days of contour integration training [124]. People with schizophrenia showed improved contour integration performance following 2–4 days of exposure to the task in the study cited above in which controls reached asymptotic performance after 2 days [80], and improved pattern recognition across a single session of training involving 600 trials [23,82]. In addition, there are numerous studies that provide validity, reliability, and short-term perceptual learning data for contour integration paradigms [8,56,124]. A further advantage of this task is that the neural mechanisms underlying performance have been demonstrated in monkeys and non-clinical human samples [125–129], and neural correlates of impairment (e.g., in V2, V3, V4, LOC, and frontal-parietal regions) have been identified in participants with schizophrenia [75,76]. Therefore, demonstration of improved performance after remediation would lead naturally to EEG and fMRI studies of training-related activation changes in specific regions of interest and brain networks.

To assess target engagement for the PO intervention (CIT), we will use two tasks. The first is the original contour integration test recommended by the CNTRICS initiative for use in treatment studies of schizophrenia [8], namely the Jittered Orientation Visual Integration (JOVI) task [8,56,76]. The JOVI involves identifying the direction of an egg-shaped contour made up of individual Gabor elements with gaps between them so that the participant has to perceptually integrate the Gabors to perceive the shape (Figure 3). The task has been optimized for use in clinical trials of people with schizophrenia [8,76]. Although there are differences between the PO training task (CIT) and the JOVI in terms of the specific stimuli (i.e., circular vs. oval shapes, respectively) and response requirements (identifying the location of the target circle vs. indicating whether the centrally-presented egg-shaped contour is pointing left or right), which should preclude confounds based on low-level perceptual learning (e.g., learning that is specific to one area of visual space, or to a single shape), we will also include a second outcome measure, one that does not share method variance with the training task. The second PO task uses the Ebbinghaus illusion, in which a center circle appears smaller if it is surrounded by (i.e., grouped with) larger circles and appears larger if it is surrounded by smaller circles (Figure 5). The task requires subjects to choose which display (on the left or right of the screen) contains the array with the larger central circle. By manipulating the difference between the actual sizes of the central circles, and whether the size of the surrounding circles causes the inner target circle to appear smaller or larger than its actual size, a psychophysically precise measure of illusion strength is obtained. The first author has used this task extensively [130–133], and one of its appealing aspects is that, due to their reduced grouping of the central target and the surrounding circles, people with schizophrenia perform *more accurately* (in all studies cited above) than controls on trials in which the surrounding context is normally misleading (e.g., when the larger of the two inner circles is made to appear smaller by surrounding it with large circles). Evidence that both the JOVI and Ebbinghaus tasks involve PO comes from a significant inverse correlation between scores such that a lower score on the JOVI is associated with higher scores in the misleading condition on the Ebbinghaus task [131].

### Study Goals, Aims, and Hypotheses

*The goal of the R61* is to determine the optimal intervention, from among two treatment strategies and their combination, for improving the targets of CS and PO. Both of these interventions, namely the contrast sensitivity training (CST) and contour integration training (CIT), are included in the Sightseeing App. We will examine the effects of these interventions individually and in combination (CST & CIT). An active computer-based control treatment (ACCT; described below) will control for non-specific training elements. We will collect data on 20 participants per condition. Each subject will complete 40 training sessions, with assessments after every 10 sessions.

#### R61 Specific Aim:

To determine the effects of CST and CIT on CS and PO targets. We will determine if treatment effects meet a pre-specified criterion for clinical significance, operationalized as a Cohen’s d (effect size) of 0.4, and if so, the minimum dose (i.e., number of training sessions) associated with this effect. Hypothesis 1a: CST or CST & CIT will lead to significantly greater gains in CS than will ACCT. Hypothesis 1b: CIT or CST & CIT will produce significantly greater improvements in PO than will ACCT. The *Go Signal* for continuing to the R33 phase is Cohen’s *d* ≥ 0.4 for Hypothesis 1a and/or Hypothesis 1b. Through the R61, we will determine which treatment and at what duration (i.e., dose) best improves the target(s); this will be considered the optimal visual remediation intervention, to be carried forward for further evaluation.

*The goal of the R33* is to conduct a randomized controlled trial (RCT) of the optimal visual remediation treatment (from the R61), testing its effects on target engagement, and on clinical outcome domains (cognition, social cognition, and functional capacity). This will be done using two parallel arms (the best R61 treatment vs. ACCT), with *n* = 50 per group.

#### R33 Specific Aims:

(1) To replicate and extend evidence for visual target engagement, using the optimal treatment from the R61, in a new and larger sample. Hypothesis 1: The optimal treatment will be more effective than the control treatment (ACCT) at improving CS and/or PO; and (2) To determine if visual target engagement is significantly associated with improvements in higher-order cognitive, social cognitive, and functional domains, and to generate estimates of effect sizes to guide future studies. Hypothesis 2: Improvements in target function will be related to changes in specific cognitive, social cognitive, and functional domains. If both hypotheses in the R33 are confirmed, the results will motivate a later RCT to assess the efficacy of a visual remediation treatment for schizophrenia with a wider range of outcome variables. If Hypothesis 1 is confirmed but Hypothesis 2 is not, the results will be used to clarify by which (other) mechanisms improvements may occur. If Hypothesis 2 is confirmed but Hypothesis 1 is not, we will evaluate if this is due to subgroup and/or state effects.

This project will advance knowledge of intervention and disease mechanisms, whether the trial results are positive or negative. In the R61, we will learn whether targeting either or both levels of visual function improves the perceptual targets. The assessment of whether changes in the visual targets drive changes in other functions in the R33 will provide information about disease mechanisms by clarifying the links between visual perception and cognitive and social cognitive function. Although there are multiple ways that a “go” signal in the R61 can be achieved, each of these possibilities would represent a novel finding in its own right and would motivate further studies of visual remediation in schizophrenia. More importantly, however, the R33 will serve, in part, as a replication and extension study of the R61: Observing that the optimal R61 intervention is effective in a second study, with a larger sample and a similar degree of target engagement, would provide confidence that any R61 findings are not spurious.

At a more basic level, as noted above, we are studying the effects of targeting basic forms of gain control and integrative processes on higher-level perceptual and cognitive processes. We are including two intervention components (CST and CIT) in order to explicitly target both CS and PO because there is evidence that both aspects of visual function are impaired in schizophrenia AND that impairments in both are related to poorer functioning in multiple domains. Because this is one of the first controlled studies of perceptual remediation of these functions in schizophrenia, we wish to remain agnostic regarding whether the combined treatment (CST & CIT) will be more effective than either CST or CIT alone, although we anticipate that the combination may have additive or synergistic effects on one or both levels of vision. By assessing improvements related to the single treatment (CST or CIT) AND to the combined intervention at each level of vision, we will be able to provide a strong initial statement regarding the important question of differential and combined intervention effects. Future clinical trials will determine whether there are subgroups of individuals with schizophrenia (e.g., those who are more impaired on CS or PO at baseline) who are especially likely to benefit from these interventions. This is not a specific aim for this initial clinical trial because even people without visual impairment can improve their visual functioning [52,60,108]; however, our data will allow us to assess the degree to which improvement is a function of baseline CS and PO.

## INNOVATION

This proposal is innovative in several respects. First, while there is a burgeoning literature on cognitive remediation in schizophrenia (e.g., [62,134–136]), much of this work targets higher cognitive processes such as executive functioning and working memory, and assumes that perception is intact. As a result, despite the large body of evidence demonstrating impaired visual perceptual function in schizophrenia, there have been few attempts to evaluate the effects of visual training on specific visual processes in this condition. While CS and PO have been shown to be plastic in terms of performance improvement with repetition in prior laboratory studies, including in people with schizophrenia [52,54,56,60,108], the effects of systematic visual training of these processes for longer than a few days had not yet been studied in this population in a controlled study at the time this grant was funded. Second, unlike many cognitive remediation interventions that use games with unclear “doses” for specific functions, we are targeting two well-understood perceptual processes with interventions that clearly target these processes [68,86,109]. Third, many current perceptual learning approaches emphasize single processing mechanisms and produce results that are specific to the trained stimulus features [124,137], which has limited generalizability. The Sightseeing program addresses these issues by combining multiple perceptual learning approaches (e.g., engagement of attention, reinforcement)—each of which has been shown in past studies to contribute to increasing the speed, magnitude, and generalizability of improved CS into an integrated perceptual learning application. A predecessor of Sightseeing, called ULTIMEYES, has been shown in non-psychiatric samples to improve not only CS but also functioning in real-world activities [52,60,108]. Fourth, the PO training program we developed (CIT) is innovative; at the time of submission of the grant application, we were able to identify only one published paper on improving PO in any population [138], and this was over 30 years old. Subsequent to beginning the project, a second paper, on PO training in schizophrenia, was published [139]. Our proposed intervention is based on knowledge gained from our 30 years of studies in controls and participants with schizophrenia regarding the factors that contribute to PO, and how performance can change over time. Despite extensive evidence for PO impairment in schizophrenia [7], little is known about the maximum extent to which it can be improved, the amount of training needed to obtain gains, the durability of gains, or their functional significance. Fifth, the additive and/or interactive effects of multiple forms of visual remediation have never been investigated. This would be the first examination of whether targeting both CS and PO is more effective than targeting either single process alone. The construct of gain control (operationalized here in the form of CS) and the construct of integration (operationalized as PO) were identified by the NIMH-sponsored CNTRICS initiative, and the RDoC cognitive domain, as high-priority cognitive neuroscience constructs relevant to schizophrenia and its treatment [68]. Sixth, we will examine training effects on higher-level processes with a focus on comparing effects on visual vs non-visual cognition, which has not yet been done in a controlled study.

## APPROACH

### Preliminary Data

#### CST pilot study:

Pilot data from patients with schizophrenia at the Nathan Kline Institute (NKI) and Rutgers showed good retention with 6 of 7 participants (86%) completing at least 30 sessions of CST. This completion rate is similar to previous single-site cognitive remediation studies [62,134,140] and to an average retention rate of 87% in a meta-analysis of 40 cognitive remediation studies in schizophrenia [136]. This is also comparable to the 93% retention rate of CST in healthy young adults carried out by the second author (co-investigator and developer of Sightseeing) and colleagues. Our preliminary work with participants with schizophrenia show that CST is well tolerated, and even enjoyed, as described in our recent publication [141]. Across our pilot-study participants, CS improved 32%, with an increase in the peak contrast spatial frequency of 1 cycle/degree (pre-training peak spatial frequency of 3.14 ± 0.24 vs post-training of 4.15 ± 0.70, *p* = 0.14, *d* = 0.96). Additionally, patients improved on contour integration (*p* = 0.11, *d* = 0.85). With such a small sample (*N* = 6), our results were not statistically significant. However, the magnitude of CS improvement that we observed is similar to that from published studies of CST with non-psychiatric samples.

#### CST findings in non-psychiatric samples:

**Figure 1.**
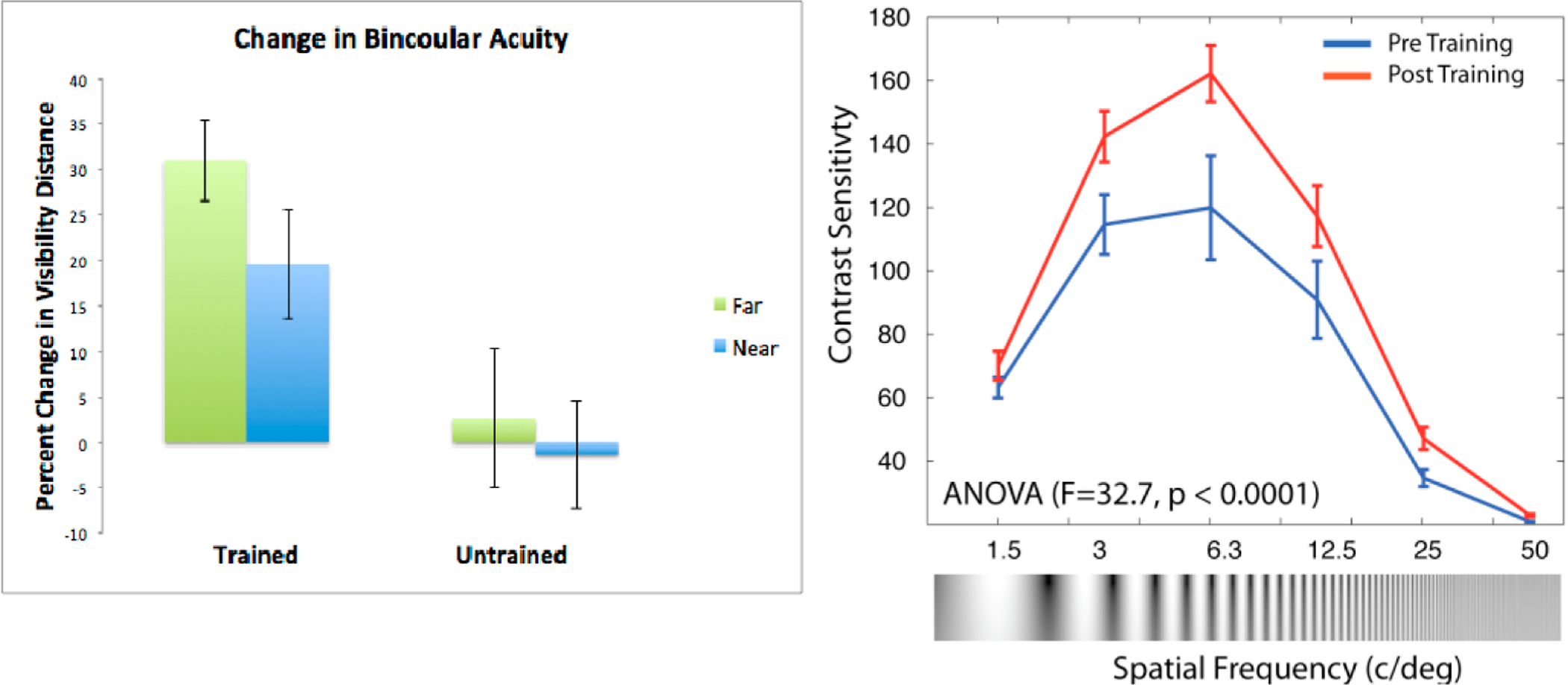
**Left:** Change in binocular acuity from pre- to post-test in CST-trained and untrained baseball players; **Right:** Change in contrast sensitivity from pre- to post training (higher scores represent better performance).

In previous research on CST conducted by the second author, CS improved in healthy normal-sighted individuals after ~30 sessions of training [108], with effect sizes ranging from *d* = 1.6 for LSF to 0.63 for HSF stimuli. In another study, college baseball players displayed significant improvements in CS (Figure 1) and batting average [60] after CST training, with effect sizes ranging from *d* = 0.34 to 0.59 (with the lowest effect size for CS to HSF stimuli). Additionally, university students demonstrated improved reading ability after CST training. Specifically, reading acuity improved an average of 13% (*d* = 0.38), moving from a pre-training mean logMAR acuity of −0.06 to a post training value of −0.11, (SD = 0.02). Reading speed improved 13% (*d* = 0.57), moving from a pre-training mean value of 240.0 words/min to a post-training value of 270.6 words/min (SD = 8.28) [52]. In addition, older adults with presbyopia displayed improved CS after undergoing CST training [52]. It is important to note that in the studies of CST in which CS was assessed [52,60,108], CS improved significantly, as seen in a shift upward across spatial frequencies (Figure 1). Effect sizes were relatively large in the trained group in these studies and the average *d* for LSFs, which is the target for this proposal, ranged from 0.45 to 1.0 across studies. Based on these data and our pilot data collected with participants with schizophrenia, as described above, we expect to observe significant CST-related gains in CS among the participants of this project.

#### Preliminary and related data that motivate the targeting of CS:

In a study conducted by the first author’s lab, we found that lower peak CS was related to poorer contour integration (*r* = 0.69, *p* < 0.01) among participants with schizophrenia (*N* = 17). The senior author’s lab recently observed that, in a sample of 32 schizophrenia participants, lower CS at 1 cycle/degree was related to poorer performance on the WAIS PO Index (*r* = 0.41, *p* = 0.02) and the MATRICS visual learning task (*r* = 0.46, *p* = 0.006). In addition, a cluster analysis indicated that participants who were more impaired on CS were also more impaired on speed of processing, PO, visual learning, and emotion recognition (*p* = 0.02 to < 0.001), but not on reasoning and problem solving or verbal learning, nor did they have more severe symptoms (*p*_s_ ≥ 0.05), suggesting that CS deficits in schizophrenia are not a manifestation of a generalized deficit [142]. These data fit current models in which CS occurs very early during visual processing, whereas PO is an integrative process that occurs later and involves binding of feature representations, the quality of which is determined in part by CS. In addition to effects of low CS on PO, studies have shown that participants with schizophrenia who have impaired CS have greater reading deficits [19,20] and poorer facial emotion detection [24]. Further, in pilot work using structural equation modeling (SEM), we have found that associations between abnormal VEPs and impaired visual learning and PO were significantly mediated by CS [143]. In short, there is reason to expect that improving both CS and PO will lead to higher-level changes in perception and cognition.

### Inclusion/Exclusion Criteria for the R61 and R33

**Inclusion**: (1) SCID-5 diagnosis of schizophrenia; (2) 18–60 years old; (3) speaks English; (4) able to complete the MATRICS Consensus Cognitive Battery (MCCB) at the baseline assessment (for the R33); (5) a raw score of 37 or greater on the Wide Range Achievement Test, Reading subtest (WRAT-3), to establish a minimum reading level (6th grade) and to estimate premorbid IQ; and (6) clinically stable as indicated by no antipsychotic medication changes in the last week or if on depot, no change in the past 1 month. **Exclusion**: (1) history of intellectual disability, developmental disorder, or neurological disorder; (2) history of brain trauma associated with loss of consciousness for >10 min or behavioral sequelae; (3) alcohol or substance use disorder within the last month; and 4) history of eye disease (e.g., glaucoma; diabetic retinopathy). Tobacco use and medication dose equivalents [144], including an index of anticholinergic load [145], will be used in data analyses.

### R61 Phase

#### Study design–R61:

80 subjects will be enrolled in this study, 20 per condition. Forty participants will be recruited at each site, namely New York Presbyterian Hospital (NYPH) and the Nathan Kline Institute (NKI). Subjects will receive 40 sessions of the intervention, and will participate in assessments of the treatment targets, CS and PO, at baseline, after every 10 sessions, and at 6 months post-training, for a total of 6 visual assessments. With 3–4 sessions per week, the intervention will be completed over 13 to 14 weeks. Each site will aim to enroll ~ 2 participants each month, which is similar to what Keefe et al. [63] estimated as a “reasonable rate of recruitment for a large-scale efficacy trial” of cognitive remediation. Given that at the 6-month follow-up, nearly all participants are expected to still be engaged in treatment in some form at NYPH or at NKI which is on the grounds of the Rockland Psychiatric Center, we expect attrition to be minimal. However, several methods, including monthly phone calls, will be used to maximize retention. For the R61 and R33, training sessions will be run in small groups by front-line staff who have already received training in these interventions, or whom we will train.

#### Treatment conditions and randomization:

To control for the amount of time spent in treatment, the four treatment conditions will consist of the following: (1) CST & ACCT (half of each session will be spent on each intervention); (2) CIT & ACCT (half of each session will be spent on each intervention); (3) CST & CIT (half of each session will be spent on each intervention; and (4) ACCT&ACCT (i.e., each session will be spent entirely on ACCT). Sessions will be the same length in each of the 4 treatment conditions. Subjects will be randomly assigned, within site, to one of the 4 conditions in a ratio of 1:1:1:1. The treatment assignment will be made after a subject has met all entry criteria and completed baseline testing, to avoid bias.

#### Go/No-Go Criterion:

We will proceed to the R33 phase if: (1) the CST-related effect size (i.e., the effect size of the difference between the CST & ACCT (or CST & CIT) and ACCT & ACCT groups in the degree of change in CS) is greater than or equal to a Cohen’s *d* of 0.4 for **either** the psychophysical **or** ssVEP CS task; and/or (2) the CIT-related effect size (i.e., the effect size of the difference between CIT & ACCT (or CST & CIT) and ACCT & ACCT groups in the change on **either** of the PO tasks is greater than or equal to *d* = 0.4. Pre-post change scores for CS and PO target assessments will be calculated for each individual and used to determine effect sizes between groups. We will not control for baseline values consistent with recommendations to use “straight” change scores when examining cognitive change across two time points [146–152].

#### Rationale for our effect size choice:

The effect size criterion we have chosen for the R61 “go” signal (*d* = 0.4) is similar to that observed in many studies of cognitive remediation, and of other treatments for this population, such as skills training and family psychoeducation [134,136,153,154]. However, less is known about effect sizes of perceptual remediation, and most of the evidence on this in schizophrenia comes from studies of auditory training using the Posit Science auditory training module [61–63], which includes training on both low-level auditory targets and higher-level auditory-verbal cognition (e.g., verbal working memory and learning) [61,155]. Results from one study indicated an improvement in the target of auditory processing speed [61] that was of large effect (*d* = 0.875). The authors also observed a medium-to-large effect on global cognition (*d* = 0.73). Results from other studies of this auditory training module in schizophrenia have indicated large treatment effects (*d* = 0.86) for both verbal learning and global cognition, and a medium effect (*d* = ~0.65) on the auditory target of P50 gating [62,155]. While these effect sizes are larger than our chosen criterion (*d* = 0.4), it is also important to note that some studies of Posit Science training modules have not demonstrated significant improvement on non-trained tasks [63,156]. Moreover, across studies, the largest effects have tended to come from studies for which daily training (5 days a week) was used, and subjects were paid for completing training sessions, and these are two conditions that can rarely be met in real-world psychiatric clinics. These considerations have informed our choice of our ‘go signal’ criterion, because there are as of yet very few published studies of visual remediation in schizophrenia. One small (*N* = 9) uncontrolled study of visual backward masking training found an effect size of *d* = 0.43 for improvement on the MATRICS visual learning task [50]. The first author’s research group observed an improvement of large effect (phi = 0.63) in contour integration performance among participants with schizophrenia after 4 consecutive days of practice on a contour integration task [80]. Even larger effect sizes were seen for changes in CS (*d* = 0.96) and PO (*d* = 0.85) in our small pilot sample of patients with schizophrenia, but given the small sample size and lack of a control group in that pilot study, it can be assumed that the real effect size is lower. Thus, findings from these initial evaluations of visual remediation support our use of *d* = 0.4 as a “Go” signal for target engagement. However, we acknowledge that it is not yet clear from the literature how an effect of this size relates to meaningful improvements in visual and higher-order cognitive processes and/or functional capacity. *The proposed R33 will allow us to address this question in a preliminary manner*. If results from the R33 trial are positive, we will seek funding for a larger trial to evaluate the impact of this treatment on functional outcomes, and to identify mediators/moderators of treatment response.

**If the Go criterion is met for any of the treatment conditions**, we will perform a thorough investigation of the effects of CST, CIT, and CST & CIT on the CS and PO target scores to determine which remediation strategy to use for the R33. Analyses will also include (i) assessment of the trajectory of performance on the target measures over the course of treatment; (ii) determinations of whether the improvements plateau prior to the 40th session or whether they continue throughout the training period; and (iii) evaluations of whether any combined effects of CST and CIT are additive or multiplicative. These goals will be accomplished by first graphically examining the trajectories of target change to assess whether they are linear, monotonic non-linear, quadratic, or any other shape. After that, appropriate models for longitudinal data analysis will be applied to estimate improvement rate and time to asymptote. We will account for CPZ-equivalent dose of antipsychotic medications in all models, using CPZ dose as a time-varying covariate if necessary, and will also account for type of antipsychotic medication and anticholinergic load. Those models will also be used to examine whether baseline characteristics moderate the effect of CST and/or CIT on the targets. These analyses will allow us to ascertain: (1) whether CST alone, by targeting CS function, has a cascading effect on PO; (2) whether CIT improves CS while targeting PO function; and (3) whether CST & CIT has a stronger effect than either intervention alone for any outcome. If all 3 treatment conditions are effective to an equivalent degree, the criterion will be reaching asymptotic level of improvement earliest.

#### Sample size determination:

The sample size of the R61 study (*n* = 20/group) was selected to ensure that when the true size of the effect of CST or CST & CIT on the CS target, or CIT or CST & CIT on the PO target, is *d* = 0.4, the 95% confidence interval (CI) for the effect size does not contain zero. Given data on the CS measure over time in the CST pilot study described earlier, a 95% confidence interval for an effect size of magnitude 0.4 is between .02 and .91. Since the observed effect size in that pilot data was actually much larger than our Go criterion (*d* = 0.96), we are confident that we will be adequately powered to detect a meaningful effect. Since participants in that pilot study also improved on a measure of contour integration (*d* = 0.85), even though training was focused only on CS (our low-level visual target), setting the effect size at *d* = 0.4 for both CS and PO seems reasonable. Note that although contour integration improved without PO training in the pilot study, this was unlikely due to practice effects on the test alone since two prior large studies did not find practice effects on the JOVI, in either healthy controls or participants with schizophrenia, over two or three repeated presentations separated by days or weeks [157,158]. Furthermore, a third study found improvements only with daily exposure to two versions of the task, and this did not occur for the schizophrenia group until the third day [56,80]. Subjects who do not complete all 40 sessions of training will be invited to complete a “post-treatment” assessment after their last session; for non-completers who decline to participate in a post-treatment evaluation, the last post-baseline assessment will be used, unless the participant dropped out before the 10th session (the time of the first post-baseline assessment), in which case his/her data will not be used in the analyses. In such cases, we will recruit additional participants to reach the target sample size. For any dropout that occurs, we expect rates to be uniform across conditions, given that we have designed the 4 conditions, including the active control, to be similarly engaging.

### R33 Phase

#### Study design–R33:

The R33 will be a two parallel-arm RCT comparing the optimal R61 treatment to the control (ACCT) treatment. One hundred subjects will be enrolled over 3 years, 50 from NKI and 50 from NYPH. Visual and clinical outcome assessments will be conducted at baseline, and at intervals determined based on R61 results, including a 6-month follow-up (with attrition considerations and procedures identical to the R61.

#### Treatment conditions and randomization:

Subjects will be randomly assigned, within site, to the optimal or control treatment in a ratio of 1:1. Treatment assignment will occur after a subject has met all entry criteria and has completed baseline testing, to avoid bias.

#### Assessments:

No matter which intervention is used for the R33, visual tests will include both tests of CS and both tests of PO used in the R61. See below for descriptions of the R33-specific cognitive, social cognitive, and functional capacity measures.

#### Data analysis:

An intention to treat approach to data analysis will be used in this clinical trial. For all analyses, statistical significance will be defined as *p* < 0.05, unless specified otherwise. Bonferroni or false discovery rate corrections will be applied to multiple testing, as appropriate. Hypothesis 1: The optimal R61 treatment will be more effective than the control treatment (ACCT) in improving CS and/or PO test scores (to be determined based on the R61 outcome). This will be tested using a linear mixed effects model in which the values of the target(s) at each time point are modeled as a function of treatment group (experimental, control), time, group x time, and potential moderators and mediators (e.g., age). If the group × time interaction is significant, we will use model-based estimation procedures to estimate the magnitude of the effect. We will also explore differential effects of the treatment on CS vs PO. Hypothesis 2: Improvements in visual processing, as observed in CS and/or PO (based on R61 results), will be related to changes on specific cognitive *(i.e., visual working memory, visual learning and memory, reading)*, social cognitive *(i.e., emotion recognition)*, and functional capacity measures. We will assess correlations between pre-post change scores for CS and/or PO target assessments and those for our cognitive, social cognitive, and functional capacity measures, and also evaluate whether changes in the target(s) either mediate or moderate treatment effects on these outcomes. An approach and computational tool described by Hayes [159] will be used to model any mediation and/or moderation effects, including indirect effects in models that involve mediation.

#### Sample size determination:

The sample size for the RCT in the R33 phase was selected to ensure sufficient power to detect medium effects of the experimental intervention on the target(s). For Aim 1 of the R33, 50 subjects per condition allows 80% power for a 2-tailed test with α = 0.05 to detect *d* = 0.57. For Aim 2, with *n* = 50 in the active perceptual training group, correlations of at least *r* = 0.38 between changes in performance on target measures (CS and/or PO) and changes in cognitive, social cognitive, and functional capacity measures can be detected with 80% power using a 2-tailed test with α = 0.05. Also, for Aim 2, *n* = 50 allows for detecting a correlation of *r* = 0.43 when making a strict Bonferroni adjustment for 3 outcomes (2-tailed α = 0.017), and *r* = 0.51 when correcting for 15 outcomes (2-tailed α = 0.003). Given the purpose of the R33 phase of this grant mechanism, our focus on effect size is exploratory and not confirmatory. We will use our observed effect sizes to power a later RCT.

### Interventions

#### Contrast Sensitivity Training (CST):

**Figure 2.**
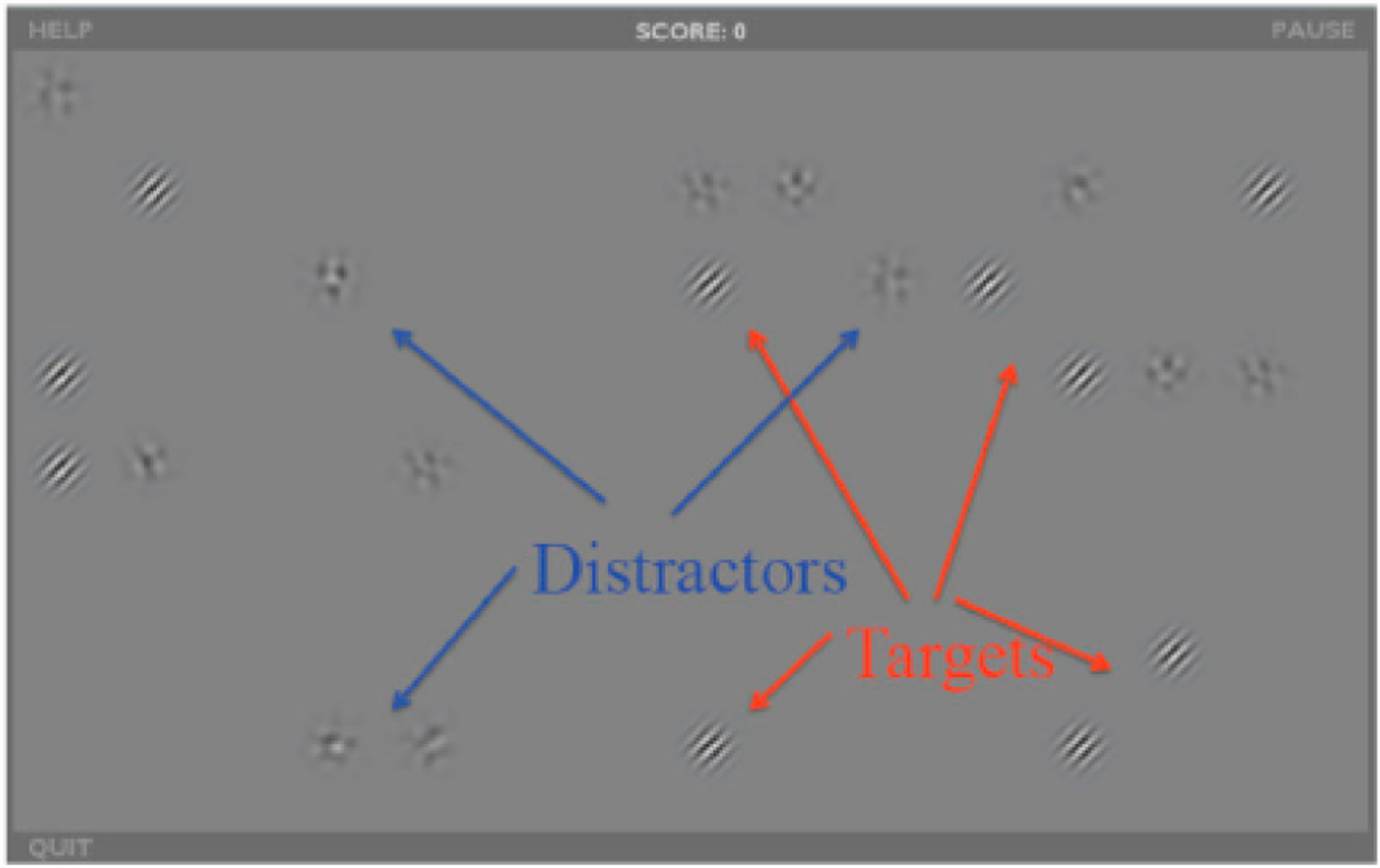
CST targets and distractors.

The CST procedure was developed by the second author and colleagues at UCR [60,108]. The program uses video game-based custom software, and the training stimuli consist of Gabor patches (game “targets”) at 6 SFs (1, 2, 4, 8, 16 and 32 cycles/degree), and 8 orientations (22.5°–337.5°). We describe this program as a “video game” because numerous elements were introduced to its design with the goal of promoting task engagement and user enjoyment. For instance, points are given each time a target is selected (and taken away when distractors are selected), and levels increase in difficulty throughout training. Contrast values are continuously tracked across sessions where each session starts off at an initial contrast for each spatial frequency that is halfway between the starting and ending contrast for that spatial frequency in the previous session. Each session consists of 8–12 training exercises that last approximately 2 min each for a total of ~25 min. The participant’s task is to click on all the Gabor targets as quickly as possible. The first few exercises consist of only targets, but distractors are added as the training progresses (Figure 2). Throughout training, distractors become more similar to the targets (starting off as blobs, then oriented patterns, then noise patches of the same SF as the targets). Targets that are not quickly selected start flickering at a 20-Hz frequency, to attract the participant’s attention [160]. At higher levels, targets and distractors appear and disappear when not selected quickly enough. Many parameters are adjusted based on ongoing participant performance, including contrast (using a 3/1 staircase for each SF), number of stimuli per trial, and presentation rate (determined by tracking average response times on prior trials for each SF). Data are saved in deidentified log files (coded with a subject number) and transmitted in an encrypted format to a HIPPA-compliant Amazon Web Services server for later data analysis.

#### Contour Integration Training (CIT):

**Figure 3.**
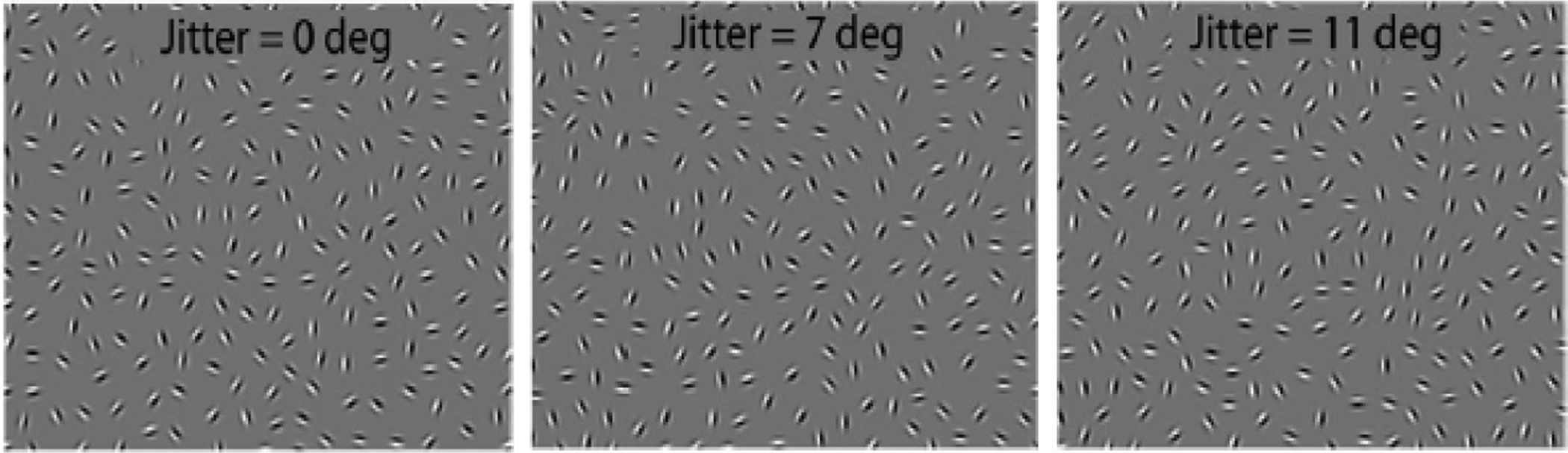
Examples of orientational jitter effects added to the egg-shaped contour from the JOVI task. This manipulation is used for circular and other CIT stimuli.

The CIT program was also developed by the second author and is based largely on two contour integration tasks we developed and have used in multiple studies of visual PO in schizophrenia [8,117]. There are two PO exercises used for this program, which are presented in alternating blocks of individual trials. Target stimuli in both exercises consist of contours that are formed by fragmented paths of individual Gabor elements, which are embedded within an array of noise Gabors. For both exercises, the participant’s task is to detect and click on the contour (e.g., a circle, oval, clover, spiral, line, curve, alphanumerical characters, etc.) formed by a set of target Gabors. For the first exercise, difficulty level is manipulated by varying the degree of orientational jitter of the Gabors making up the target contour (see Figure 3), which is done within block, and by varying the number of elements that make up the contour, which is done between blocks. The degree of jitter is determined adaptively using a “3 up, 1 down” staircase at steps of 1 degree; jitter values and element density were chosen based on data from multiple previous studies with participants with schizophrenia and controls [8,56,76,161]. Use of a staircase procedure is designed to drive performance gains, and to continuously challenge participants while ensuring continued success. For the second exercise the number of inducers of the contour are reduced via a staircase to increase task difficulty over time. Several types of contours are included to promote generalization, including those involving shapes, alphanumeric characters, and open contours such as lines, curves and spirals. For both exercises, the arrays of Gabor elements have a peak SF of 4 cycles/degree (to eliminate potential effects related to impairments in processing LSF information) and a Gaussian envelope SD (space constant) of 7.3 arcmin. Like CST, CIT is presented as a game: Participants are provided with feedback about their response accuracy, points are given for each correct response, and positive feedback is provided when participants progress to the next difficulty level. Each session consists of 8–12 training exercises that last approximately 2 min each for a total of ~25 min. As with CST, data are saved in deidentified log files (coded with a subject number) and transmitted in an encrypted format to a HIPPA-compliant Amazon Web Services server for later data analysis.

#### Active Computer-Based Control Treatment (ACCT):

Our control condition, Happy Neuron™, is a cognitively challenging remediation program that does not specifically target perception. Happy Neuron is an online brain training application that targets multiple domains of cognition. For this project, we wished to avoid any modules that focused specifically on vision or visual attention. Therefore, although Happy Neuron can be personalized to a high degree, for this study training exercises were limited to “Catch the Ladybug”, “Towers of Hanoi”, and “Elephant Memory”, which involve speed of processing, executive function, and verbal memory, respectively.

#### Tracking of Performance for Patients:

A notebook was made for each patient. At the end of each training session the CST and CIT programs show a screen with the score, level of training attained, number of errors, and whether performance earned them a gold, silver, or bronze “virtual” medal for the day. This was logged in the notebook each day so the patient could see how much they had improved over time. The “trainers” went over this with the patients each day. A similar procedure was done with Happy Neuron with the level, average accuracy, and average time logged daily for the three activities.

## ASSESSMENTS

### Diagnostic and Clinical Assessments for the R61 and R33

#### Diagnosis:

The Structured Clinical Interview for DSM-5 (SCID-5) [162] and all available clinical information will be used to assign a consensus diagnosis.

#### Verbal IQ estimate:

WRAT-III, reading subtest [163]. This task involves participants reading and pronouncing aloud a list of words.

#### Symptoms:

The Positive and Negative Syndrome Scale (PANSS) assesses the presence and severity of symptoms commonly found in schizophrenia; it is conducted as a semi-structured interview. There are a total of 30 items [164].

## VISUAL TARGETS

### Contrast Sensitivity:

The psychophysical CS assessment will be performed using an EvokeDx device, which utilizes an organic LED display that enables accurate linearization of the voltage-to-luminance relationship through customized gamma correction so that precise specification of contrast can be achieved. These features, in addition to the carefully calibrated amplifiers contained in the system, afford high reliability/reproducibility of stimulus presentation across multiple EvokeDx devices, which is critical when testing at multiple sites. The same stimulus parameters and testing conditions will be used at the NYPH and NKI sites, and automated luminance calibration will be performed monthly at each site using the photometric device provided with the EvokeDx by Konan Medical (Irvine, CA). Amplifier settings are as follows: gain = 20 K, bandpass filter = 0.5–100 Hz. An earlier version of the system now provided by Konan equipment for assessing CS was successfully used in a prior multi-site trial [165].

CS functions will be obtained by presenting 2 horizontal sine-wave gratings at spatial frequencies of 0.4 and 6.5 cycles per degree. Spatial frequency is the number of pairs or cycles of light and dark bars in 1 degree of visual angle, expressed as cycles/degree, with fewer pairs corresponding to lower SF. Gratings of 0.4 cycles per degree (LSF) will be presented for 33 ms and gratings of 6.5 cycles per degree (HSF) will be presented for 500 ms. An up-and-down transformed response method will be used to obtain contrast thresholds with a criterion of 70.7% correct responses for each SF. Ten reversals are obtained and the mean of the last 5 reversals will be used to obtain thresholds. A spatial 2-alternative forced-choice procedure will be used. Gratings will be presented on either the right or left side of the screen, and the participant’s task is to determine on which side the gratings appeared. Results will be plotted as CS (which is the reciprocal of threshold) vs SF. Increased CS indicates better performance. Participants will be tested binocularly after being light-adapted to the background luminance of the display for 15 min. Test retest reliability of this measure was evaluated by the senior author’s lab in a group of 15 controls and 31 participants with schizophrenia; the ICC was 0.76 at 0.5 cpd and 0.67 at 1 cpd. The test-retest reliability is weaker (0.25) at the SF of 4 cpd that produces the highest a CS but improves with higher SFs (ICC = 0.69 for 7 cpd and 0.57 for 21 cpd). The target variable is the CS for the SFs of 0.4 cpd and 6.5 cpd. We expect to see more of an effect of the remediation on LSF, but we will also assess CS at the higher SF, and specifically compare LSF results to HSF results, which also have high reliability, to determine if effects are LSF-specific.

### VEP Contrast Responses:

**Figure 4.**
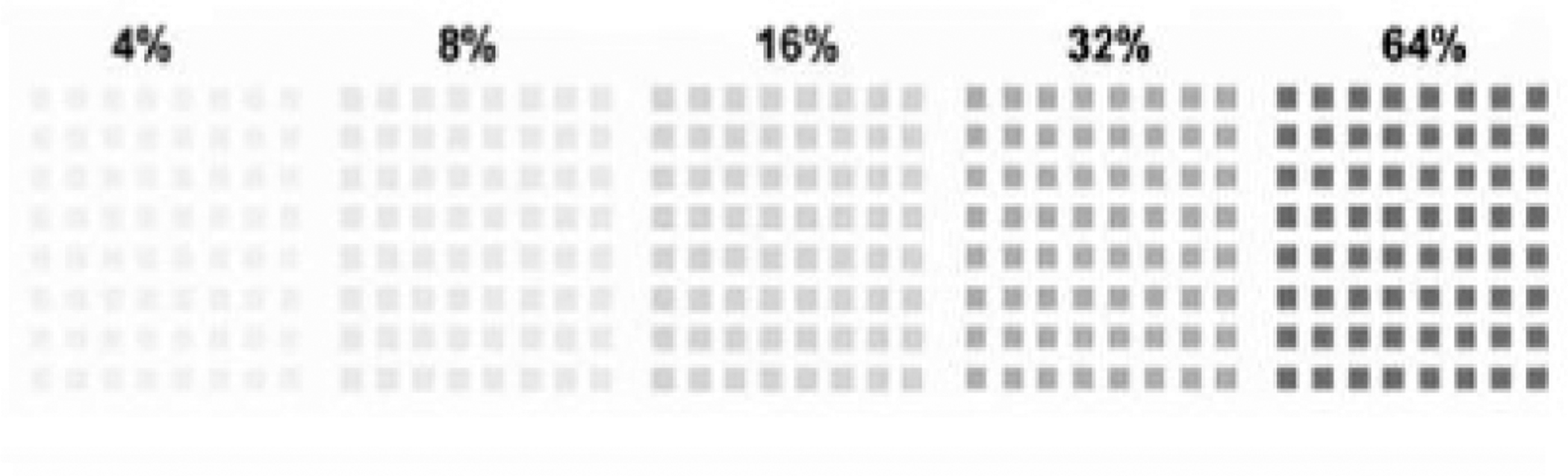
VEP contrast stimuli examples.

VEPs will be obtained using the EvokeDx device, with the active electrode over the occipital lobe (Oz). EvokeDx has FDA 510(k) clearance for assessment of visual neural function. It generates the stimuli, records and analyzes the electrophysiological signals, and stores the data. The VEP technique we will use was developed by Zemon and colleagues [96], and has been used in studies of schizophrenia [1,24,69], autism [95], and glaucoma detection [165]. The response measures are quickly and easily obtained, requiring no behavioral response from the participant. Parameters to be used have been optimized in our studies of schizophrenia [1,93]. Steady-state VEPs are elicited to checkerboard patterns (Figure 4) that are luminance-modulated sinusoidally (~12 Hz) with contrast increases in 7 discrete octave steps. Each step is ~1.6 s in duration to yield an entire contrast-response function in less than 10 s. The initial step has 0% depth of modulation (DOM), and this is followed by steps of 1, 2, 4, 8, 16, and 32% DOM. The set of steps is presented 10 times. In the contrast response function, as the DOM rises, the signal-to-noise ratio (SNR) increases from below a value of 1 to a value greater than 1. Test-retest reliability of low-contrast VEP responses in schizophrenia is good (ICC = 0.70, *N* = 32; unpublished data of the senior author’s lab). In addition, the 95% confidence regions for the 10 runs per person show good reliability within an individual [96]. Furthermore, the senior author has observed a within-subjects correlation of *r* = 0.41, (*p* < 0.001, *N* = 74) between indices from these VEP and psychophysical CS assessments.

### Jittered Orientation Visual Integration (JOVI) Task [8,75,76]:

For this PO task, stimuli consist of oval contours, made up of 18 Gabor elements separated by 1° of visual angle, that either point left or right (Figure 3). The contours are embedded in 298 distractor Gabors. Difficulty is manipulated by increasing the degree of orientational jitter of the Gabors making up the contour. Jitter levels will be 0°, 7°, 9°, 11°, 13°, and 15°, as in recent studies. Trials will be blocked according to the amount of orientational jitter, with 12 trials per block. In addition, each block will contain 4 catch trials in which a contour with no orientational jitter is presented without background elements, or a contour is presented with background elements but with a line drawn along the contour. These trials are included to identify subjects who respond randomly or who are not paying adequate attention to the task. As in past studies, only subjects who obtain 75% or higher accuracy on these trials will be included in data analyses. Blocks will be presented in increasing order of difficulty, with each block presented 4 times for a total of 384 trials (4 repetitions × 6 blocks × 16 (12 regular, 4 catch) trials). Each stimulus is shown for 2 s, followed by a 1 second inter-stimulus interval. The participant presses a right or left arrow key to indicate the direction of the contour. This task was optimized for use with participants with schizophrenia in a previous 5-site study [8], which found good test-retest reliability [157]. The dependent variable is number correct, corrected for guessing.

**Figure 5.**
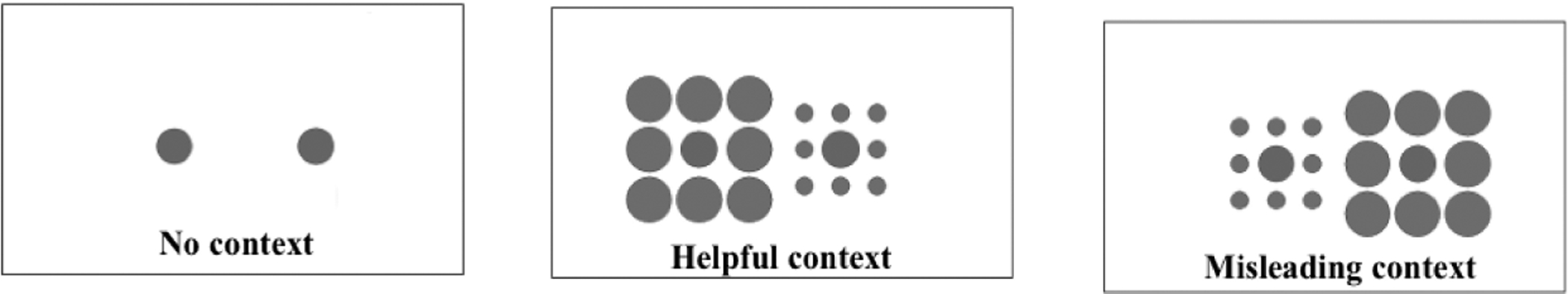
Examples of the 3 Ebbinghaus task conditions.

The Ebbinghaus Illusion Task [132,166–168] is our second measure of PO. On each experimental trial, subjects are shown two target circles - one on the left of the screen and one on the right, and their task is to indicate which is larger. On half the trials, these circles are presented by themselves (i.e., the no-context condition). On the other half, the targets are surrounded by larger or smaller circles that either *facilitate* perceiving the true size difference of the target circles (in the helpful condition), or *hinder* perceiving the true size difference (in the misleading condition). In the *helpful condition*, the larger inner circle is surrounded by smaller circles, making it appear larger than its actual size, and the smaller inner circle is surrounded by larger circles, making it appear smaller than its actual size; these effects combine to amplify the real size difference between the target circles. In the *misleading condition*, the larger inner circle is surrounded by even larger circles, making it appear smaller than its actual size, and the smaller inner circle is surrounded by even smaller circles, making it appear larger than its actual size. For example, see Figure 5, where the target circle on the right is 2% larger in each of the 3 panels. Stimuli remain on the screen until the subject responds or for 2 s (whichever occurs first). If a response is not recorded within 2 s, the trial is recorded as a guess (0.5 correct). Trials are separated by 200 ms. The two target circles always differ in actual size and this size difference varies in magnitude across trials. The order of trial types is randomized for each subject, as is the side on which the larger inner circle appears on each trial. In total, the task contains 192 trials, and typically takes approximately 7 minutes. The key metric from this task is the difference between the helpful and misleading conditions, controlling for no-context performance, or: [(Helpful—no context)-(misleading—no context)]. Reduced grouping is reflected in scores closer to zero.

### Visual Acuity:

While not a target for this study, acuity will be assessed to determine whether it moderates the effects of CST and/or CIT [4,169]. We will use standard high-contrast ETDRS charts, which are the “gold standard” for acuity testing [170], along with Sloan low-contrast letter acuity charts [171] to assess low-contrast vision.

## OTHER OUTCOME MEASURES FOR THE R33

### MATRICS Consensus Cognitive Battery (MCCB):

The MCCB assesses multiple cognitive domains, namely Speed of Processing, Attention/Vigilance, Working Memory (visual and verbal), Verbal Learning, Visual Learning, Reasoning and Problem Solving, and Social Cognition [172,173]. The ICC for the MCCB composite score was 0.9 in the initial validation study and has been similarly high in multisite clinical trials [173–176]. Outcomes are T-scores for each of the domains and the composite score [172,173]. Exploratory analyses will include assessing whether treatment effects on **visual** working memory and **visual** learning are stronger than for non-visual memory and learning subtests.

### Minnesota Low-Vision Reading Test (MNREAD):

This test assesses reading speed ability. The charts contain 19 English sentences (60 characters each) with print sizes ranging from 1.3 to −0.5 logMAR at a distance of 16 inches (0.41 m). Participants are instructed to read each sentence aloud as quickly and as accurately as possible. Outcomes are reading acuity, speed, and critical print size. The MNREAD is resistant to practice effects and has strong test-retest reliability [177].

### Penn Emotion Recognition Test (ER-40):

This computerized task comprises 40 photographs of actors expressing one of 4 basic emotions (happiness, sadness, anger, fear) or a neutral expression [178]. Participants are asked to select, from these choices, which emotion is being expressed for each image. The outcome variable is total percent correct. The ER-40 has been used widely in schizophrenia research, including in multi-site studies (e.g., [179]). The ER-40 demonstrates sound convergent and discriminant validity and good test-retest reliability in participants with schizophrenia (ICC = 0.75 [180]).

### University of California, San Diego Performance Based Skills Assessment, 2nd Edition (UPSA-2):

This is a performance-based measure of the extent to which participants are capable of performing specific living skills such as household chores, communication, finance, transportation, and planning recreational activities [181]. We will use the total score, which ranges from 0 to 100. Test-retest reliability is 0.63–0.80 over periods of up to 36 months [182]. UPSA scores significantly predict residential independence [183,184].

## SUMMARY AND UPDATE

The goals of this project are: (1) in the R61, to establish the optimal components and dose of a visual remediation intervention from among two specific visual training strategies, and their combination, for improving low and mid-level visual functions in schizophrenia; and (2), in the R33, to determine the extent to which this “optimal intervention” improves not only visual processing but also higher-level cognitive and role functions. At the time of writing this paper, we have completed data collection for the R61 phase and data analyses are underway. A later report will describe the R61 results. Our preliminary data, including the early results from the current R61, along with a recently published study on remediation of PO in schizophrenia [139], all suggest that training of visual functions in people with schizophrenia is both possible and beneficial. The final results of this study will allow for conclusions about the durability and generalizability of these benefits, and about potential mediators and moderators of treatment effects. A long-term task is to determine whether combining lower-level sensory and perceptual training with higher-level cognitive training (the level typically targeted in research and clinical practice) leads to even greater gains in functioning than are observed using currently available cognitive remediation interventions.
